# Efficiency of an Ultrafiltration Process for the Depollution of Pretreated Olive Mill Wastewater

**DOI:** 10.3390/membranes15030067

**Published:** 2025-02-20

**Authors:** Mohammed Zine, Noureddine Touach, El Mostapha Lotfi, Philippe Moulin

**Affiliations:** 1 Laboratory of Spectroscopy, Molecular Modeling, Materials, Nanomaterials, Water and Environment, Environmental Materials Team, ENSAM, Mohammed V University in Rabat, Avenue des Forces Armées Royales, Rabat B.P. 6207, Morocco; mohammedzine34@gmail.com (M.Z.); nour-eddine.touach@ensam.um5.ac.ma (N.T.); el-mostapha.lotfi@ensam.um5.ac.ma (E.M.L.); 2 Aix Marseille Univ., CNRS, Centrale Méditerranée, M2P2 UMR 7340, Équipe Procédés Membranaires (EPM), Europole de l’Arbois, BP80, Pavillon Laennec, Hall C, 13545 Aix en Provence, CEDEX, France

**Keywords:** olive mill wastewater, ultrafiltration, hybrid process, MWCO effect

## Abstract

The depollution of constructed wetland-pretreated olive mill wastewater (OMW) using a membrane filtration system was experimentally studied. Dead-end filtration (DEF) was employed to evaluate suitable MF/UF membranes and select the appropriate molecular weight cut-off for optimal OMW treatment. Removal efficiencies for COD (chemical oxygen demand) and TOC (total organic carbon) using DEF reached maximum values of 88.14% and 11.17%, respectively. Adsorption of raw and pretreated OMW on granular activated carbon was also carried out for a comparative study against DEF and pretreatment. The semi-industrial-scale experiments were conducted using commercial ceramic ultrafiltration (UF) membranes (150 and 50 kDa) in cross-flow filtration (CFF) mode at a permeate flux around 200 L h^−1^ m^−2^ and a trans-membrane pressure (TMP) of 3.5–3.8 bars. This post-treatment had a significant impact on COD removal efficiency from pretreated OMW, reaching 78.5%. The coupled process proposed in this study achieved removal efficiencies of 97%, 97%, and 99.9% of COD, TOC, and turbidity, respectively.

## 1. Introduction

The increase in olive oil production has led to the excessive generation of liquid by-products, commonly known as olive mill wastewaters (OMWs), which represents a major issue for the olive industry. It is estimated that 107 m^3^ of these wastes are produced each year worldwide, most of them in the Mediterranean region. Reducing the volume generated annually poses a significant environmental challenge for the countries in this region [1,2].

OMWs have an acidic reaction and appear as dark liquid effluents. Their pH ranges from 4.5 to 5.9 [3,4]. The main cause of the pollution load of this wastewater is its high content of organic matter, particularly polyphenols. The chemical oxygen demand (COD) and biochemical oxygen demand (BOD_5_) of OMWs can reach 220 g L^−1^ and 100 g L^−1^, respectively. Phenolic compounds, whose concentration can vary from 0.5 to 24 g L^−1^, are responsible for the high resistance of vegetation water to biodegradation and phytotoxicity [4,5].

To date, Morocco has no specific legislation governing the management of these liquid effluents. Most Moroccan and international oil mills discharge their OMWs into natural evaporation basins. The long storage time in these basins can be considered as a natural biological treatment process. However, this method poses several problems, including the release of foul odors, greenhouse gas emissions, infiltration into the soil, and the multiplication of insects. The high concentration of oily residues in these wastewaters also promotes the formation of films that cover the ponds, thereby reducing natural evaporation. Some Moroccan oil mills discharge their effluent into the environment without prior treatment, resulting in significant contamination. The continuing increase in both domestic and foreign demand for olives and olive oil will worsen this issue in the coming years [1,6].

Research is currently focused on developing biological and technological applications for the generation of significant metabolites and recovering fine from OMWs. By recycling and reusing resources, this strategy aims to close the production loop in accordance with the principles of circular economy, benefiting the economy, the society, and the environment [5,7].

The constructed wetlands (CWs) present a viable alternative to wastewater treatment and pre-treatment. The low energy consumption of these systems is especially useful in places with limited access to reliable energy sources. Additionally, CWs are typically more cost-effective to build and maintain, due to the use of locally available materials and the reduced requirement for complex mechanical systems. This economic advantage is particularly important for small communities with limited financial resources. CWs consist of plants, soils, and microbial communities that treat wastewater through a combination of biological (e.g., biomass attached to substrate media), chemical (e.g., adsorption, complexation), and physical (e.g., filtration) processes. As a result, CWs are highly effective in reducing COD, suspended solids, heavy metals, and pathogens [8,9,10]. Fleyfel et al. [11] reported that during the first coagulation/flocculation step, the application of 8 g L^−1^ of lime combined with 7 g L^−1^ of aluminum sulfate (alum) achieved removal rates of approximately 10% for electrical conductivity (EC), 41% for total solids (TS), and 48% for COD. In the second coagulation/flocculation step, the use of 5 g L^−1^ of lime and 4 g L^−1^ of alum resulted in lower reduction rates for TS (37%) but higher reduction rates for COD (67%). Additionally, Vaz et al. [12] emphasized that a sedimentation for 12 h resulted in removal rates of 45%, 50%, and 64% for TOC, COD, and BOD_5_, respectively. Moreover, a combined process of coagulation followed by ultrafiltration achieved removal rates of 80% for TSS and 90% for COD. CWs are typically classified based on the type of macrophyte utilized (emergent, submerged, or free-floating) and the wastewater flow regime, which can either be free water-surface flow (FWSF) or subsurface flow (SSF). The SSF category is further subdivided according to the direction of the flow path; the two main types are horizontal flow and vertical flow. In vertical flow systems, wastewater percolates vertically through the bed, facilitating drainage toward the system’s outlet. The outlet enables the possibility of incorporating an additional finishing treatment, as presented in this study, using ultrafiltration. The vertical percolation process also promotes the refilling of the bed with air, thereby enhancing oxygen transfer into the substrate. The increased oxygen availability supports aerobic processes such as nitrification and contributes to the effective removal of organic matter. These systems generally consist of a flat bed of sand and gravel, planted with vegetation, with treated effluent collected through a drainage system at the outlet. As a result, vertical flow systems have gained growing interest and have been the subject of extensive research over time. However, this system category does not provide optimal conditions for denitrification. As a result, gaseous nitrogen compounds, which would typically be released into the atmosphere, are not produced. Consequently, ammonia nitrogen (ammonia-N) is predominantly converted into nitrate nitrogen (nitrate-N) [8].

Over the past few decades, membrane processes have emerged as effective methods for enhancing the valorization of agro-food wastewaters and by-products in sustainable biorefinery frameworks. These processes provide numerous benefits compared to traditional separation techniques, including mild temperature and pressure conditions, straightforward equipment, easy scaling, low energy consumption, no use of chemicals, and high selectivity for specific compounds [5,13].

Membrane processes have been extensively studied to produce effluents of acceptable quality from OMWs for environmentally safe disposal. Various valorization strategies have also been proposed, combining membrane-based operations in a sequential manner to recover, fractionate, and concentrate phenolic compounds from these effluents. Most of this research focuses on pressure-driven membrane techniques, including microfiltration (MF), ultrafiltration (UF), nanofiltration, and reverse osmosis [5,14]. Microfiltration (MF) and ultrafiltration (UF) have emerged as effective alternatives to traditional industrial separation methods. They offer several advantages, including high selectivity, efficient oil removal, ease of maintenance, continuous and automated operation, cost-effectiveness, low energy consumption, and compact modular designs [15,16,17]. Cifuentes-Cabezas et al. [18] treated OMWs using ceramic membranes with molecular weight cut-offs of 5, 15, and 50 kDa. Under operational conditions of 3 m s^−1^ and 3 bar, the 15 kDa membrane achieved significant removal of color (72%) and turbidity (99%), while reducing the organic load by 54%. It seems that ultrafiltration alone does not achieve a very high COD rejection rate, typically around 48% to 63%: A hybrid process is necessary to achieve higher rejection rates [19,20]. For example, Ouadah et al. [21] developed an integrated hybrid process for the OMW treatment by incorporating an ultrasonication step in combination with UF. Their approach achieved removal efficiencies of 88% for COD, 85% for TOC, 74% for polyphenols, and 91% for ammonium nitrogen. Additionally, the process successfully eliminated microorganisms, including fungi and sulfite-reducing anaerobes, as well as fatty acids [22,23,24]. The application of ultrasounds induced changes in the dynamic properties, such as OMW’s viscosity, which contributed to an enhanced permeate flux through the UF membrane [21]. Saf et al. [25] conducted a comprehensive analysis of fouling phenomena in a 150 kDa ceramic tubular membrane used for OMW ultrafiltration. At three different pH levels (2, 6, and 9), the membrane exhibited a high retention of suspended solids (98%), while retaining only 1% of phenolic compounds. At pH 2, the system achieved a modest volume concentration ratio (VCR) of 15%, along with a low permeate flux of 15 L h^−1^ m^−2^. This condition was characterized by the formation of a compact, highly mineralized pectin gel layer, which resulted in significant membrane fouling. In contrast, at pH levels 6 and 9, the productivity remained similar, with an initial permeate flux of 160 L h^−1^ m^−2^ and a final VCR of 80%. Moreover, UF at pH 6 was associated with reversible fouling, primarily due to the accumulation of pectin gel and agglomerates of suspended matter. In another study, Cifuentes-Cabezas et al. [26] proposed the forward osmosis (FO) process as a viable method for concentrating phenolic compounds from OMWs for subsequent recovery. The two membranes used in their study effectively concentrated phenolic compounds, achieving acceptable VCR values. Notably, higher concentrations of total phenolic content were observed in the FO tests compared to those using NaCl as a draw solution, with values ranging from 74.13% to 76.93%, compared to 62.6% in the NaCl-based tests. Although UF tests exhibited higher flux, the recovery percentages of total phenolic content were similar across both processes. Several studies focus on a few recent processes (simple and coupled), demonstrating that a single treatment process cannot achieve high COD separation efficiency [12].

In this study, a hybrid process combining CW oxidation followed by membrane filtration was developed to treat OMWs. Dead-end filtrations are carried out to estimate the molecular weight cut-off of the cross-flow semi-industrial-scale experiments which concentrate OMWs and produce purified effluent. Additionally, granular activated carbon experiments are performed to compare the different stages of the developed process.

## 2. Materials and Methods

### 2.1. Description of the Constructed Wetland System

Two CW units were situated outdoors, with a surface area of 1 m^2^, a depth of 60 cm, and a slope of 1% along their lengths. The units were filled with a layered composition of coarse gravel, fine gravel, and sand. The first layer, consisting of 10 cm of coarse gravel with a particle diameter ranging from 20 to 40 mm, was placed at the distribution tap to facilitate the passage of the effluent. Above this, a 10 cm layer of fine gravel (particle diameter ranging from 2 to 8 mm) was installed. The third filter layer was composed of 30 cm of sand. The last layer, composed of 20 cm of coarse gravel (with a diameter between 20 and 40 mm), was placed at the top to avoid odors and wastewater evapotranspiration. Prior to entering the treatment units, the influent wastewater was processed in a 2 m^3^ settling tank, which provided a 24 h retention time. This step improved influent quality by removing suspended particles, thus reducing the risk of clogging. The units were uniformly supplied with 25 L of wastewater every four hours by an electric pump operating in parallel [8].

**Figure 1:**
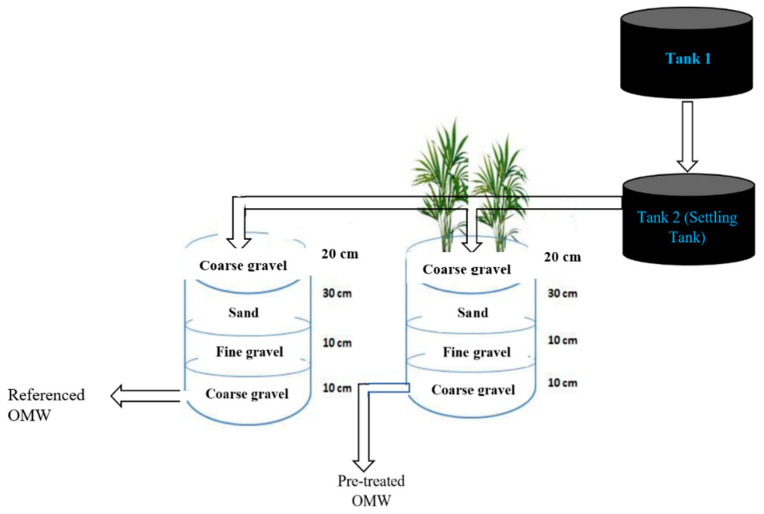
Schematic representation of the CWs units.

The experimental system comprised two beds. The first bed was planted by Phragmites Australis and the second bed was left unplanted to serve as a control, enabling the investigation of the vegetation’s efficiency and role. The plant utilized had a planting density of five rhizomes in the vegetated bed. As illustrated in Figure 1, Tank 1 was consistently filled with OMW from the Rabat-Temara region every 24 h. In contrast, Tank 2 served as the settling tank. This tank provided a continuous feed to the two CW units, delivering 25 L per unit every 4 h, resulting in a total of 150 L per day per unit [8].

Once the biocenosis had developed and the system reached a relatively stable state, as evidenced by consistent and reproducible analytical results, wastewater samples were collected twice daily. The samples were transported in plastic bottles within an icebox maintained at 4 °C and were promptly transferred to the laboratory for analysis [8].

To assess the quality of wastewater before and after pre-treatment, a range of physicochemical parameters were measured, including pH, electrical conductivity (EC), total suspended solids (TSS), turbidity, COD, biochemical oxygen demand (BOD_5_), ammonium, and orthophosphates. Table 1 presents the characteristics of the settling tank effluent. The OMW settled for 24 h in a 2 m^3^ settling tank prior to being fed into the planted and unplanted units. This step aimed to enhance wastewater quality by removing suspended particles, thereby preventing clogging of the units. The characteristics of the effluent from the settling tank are presented in Table 1. The removal rate (RR) for each parameter was calculated.

COD, TSS, and BOD_5_ concentrations in the outlet of the settling tank are 50,850, 25,233, and 22,099 mg L^−1^, respectively, which correspond to reductions of 50, 53, and 47%. Removal rates for BOD_5_ and TSS can exceed 65% and 70%, respectively, as reported in [27]. Sedimentation and microbial degradation are the primary processes responsible for the removal of COD, BOD_5_, and TSS in the settling tank. The efficiency of these removal processes is influenced by both the quality of the influent and the pattern of wastewater inflow. However, despite the effective performance of the settling tank, the effluent feeding the CW units still contained high concentrations of COD, BOD_5_, and TSS, as well as elevated levels of NH^4+^ (341 mg L^−1^) and PO_4_^3−^ (919 mg L^−1^). Wastewater turbidity was reduced by 40%, TOC by 48%, and as expected, pH and electrical conductivity values remained almost the same.

CWs are commonly regarded as an effective method for mitigating wastewater contaminants due to their low construction and operational costs. Table 1 compares the two VFCW unit removal rates (RR) of COD, BOD_5_, NH_4_^+^, and PO_4_^3−^, relative to the settling tank effluent. The two VFCW systems studied demonstrated significant pretreatment capacity. Despite receiving effluent with a high pollutant load, the systems achieved notable reductions in contaminants.

The comparison study between unplanted and planted CW units showed that all pollutants removal rates were high in the planted one, which achieved maximal removal rates of 78%, 82%, and 94% for COD, BOD_5_, and TOC, respectively. The unplanted unit delivered the lowest removal rates of 72%, 69%, and 92% for COD, BOD_5_, and TOC, respectively. This finding underscores the significant role of vegetation in pollutant degradation, thereby enhancing pretreatment performance. The presence of plants improves oxygenation within the system, and their root structures provide an ideal environment for bacterial colonization. This observation is consistent with studies by [28,29], which demonstrated that species such as Typha latifolia, Juncus subulatus, and Arundo donax in CWs enhance porosity. The rapid development of roots and rhizomes facilitates water percolation, preventing clogging. In contrast, unplanted soil exhibited clogging issues during the winter months throughout the duration of the experiment. The primary function of plant roots is to maintain consistent permeability and to create a rhizosphere conducive to the development of aerobic bacteria. Research has shown that plant root secretions influence both the quantity and diversity of microorganisms within the rhizosphere [30]. However, the significant removal rates observed in the unplanted unit suggest that the substrate also contributes to removal efficiency. Generally, microorganisms degrade organic matter in the upper soil layers, where oxygen-rich conditions prevail. Therefore, it can be concluded that the removal of organic materials is primarily driven by physical processes such as sedimentation and filtration, in addition to biological processes involving the microbial community and higher plants [28]. The treatment described in the section below was carried out on the pretreated OMW, which showed lower concentrations for COD, BOD_5_, TOC, ammonium, and orthophosphates.

### 2.2. Membrane and Adsorption on Activated Carbon Processes

#### 2.2.1. Effect of the Molecular Weight Cut off

Dead-end filtration (DEF) is a valuable method for evaluating suitable MF/UF membranes and selecting the appropriate molecular weight cut-off for optimal OMW treatment, based on permeate flux. The DEF experiments were conducted using an Amicon stirring cell equipped with a flat membrane with an area of approximately 1.73 × 10^−3^ cm^2^. The tests were performed at a transmembrane pressure (TMP) of 1 bar for MF and 3 bar for UF, with stirring using a magnetic bar to minimize the membrane fouling. Flat membranes from a Millipore brand were tested, with pore sizes ranging from 100 to 0.1 µm for MF and molecular weight cut-offs (MWCO) ranging from 1 kDa to 100 kDa for UF. Three independent experiments for the same MWCO were performed, with a new membrane used for each filtration. The three samples from each experiment were collected for analysis of COD, TOC, turbidity, pH, and conductivity, and average values were registered. The mean absolute error (MAE) was calculated as follows:

$$MAE=\frac{\sum |x_{i}-X|\;}{N}$$

where $x_{i}$ is the measured value, *X* is the mean of measured values, and *N* represents the number of values.

#### 2.2.2. Cross-Flow Filtration (CFF)

**Figure 2:**
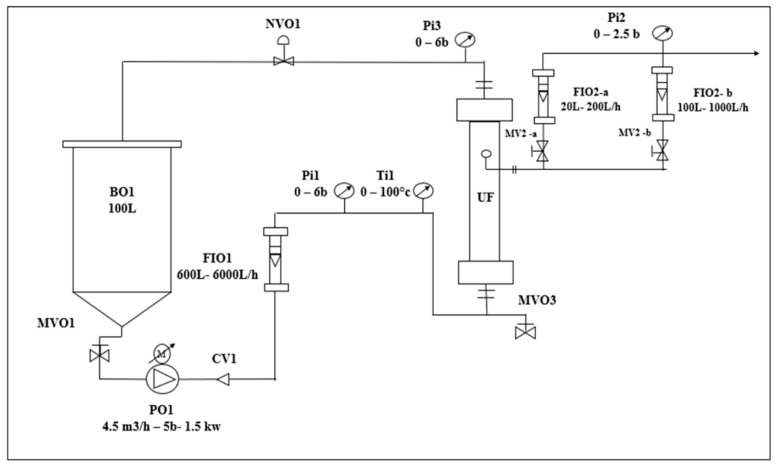
CFF pilot plant (BO, feed tank, PO pump, FIO, flowmeter, Pi manometers, Ti thermometer, and UF module).

Cross-flow filtration (CFF) is the preferred industrial membrane filtration method when dealing with OMW. In this study, ceramic tubular ultrafiltration (UF) membranes with 19 channels (Alsys society, Salindres, France) and a molecular weight cut-off of 150 and 50 kDa were used, providing an effective filtration area of 0.25 m^2^. The pilot plant is presented in Figure 2.

The wastewater was stored in a 100 L feed tank and circulated through a stainless-steel membrane module via a centrifugal pump. Initial water permeability (Lp) (L h^−1^ m^−2^ bar^−1^) was calculated as the slope of the linear regression between transmembrane pressure (TMP) and the permeate flux J at 20 °C. During UF experiments, samples of feed wastewater, retentate, and permeate were collected to analyze the retention rate and the mass balances.

#### 2.2.3. Membrane Fouling and Washing

Membrane UF fouling during OMW filtration occurs due to the deposition of organic matter on the membrane surface, or through strong physical or chemical adsorption onto the membrane itself, leading to a flux decline. The initial flux can be recovered by cleaning with alkaline and acidic solutions. Gruskevica et al. [31] highlighted that in large-scale systems, the use of strong alkaline reagents, first, is a common practice for membrane cleaning, as they effectively dissolve organic foulants. Among these reagents, sodium hydroxide (NaOH) is preferred over potassium hydroxide (KOH) due to its superior solubility, higher cleaning efficiency, and lower cost. Typically, NaOH is used at concentrations ranging from 0.5 to 2% by weight. Alkaline cleaning is typically followed by acid cleaning to address inorganic fouling. Acids are effective in dissolving inorganic sediments, such as metal oxides and encrusted proteins. Nitric acid (HNO_3_) is most commonly employed for this purpose. HNO_3_ is employed with typical concentrations ranging from 0.5% to 1.5% by weight [32]. In this study, chemical cleaning of the fouled membranes was performed after each experiment, following these steps: (i) washing with sodium hydroxide (20 g L^−1^) at 80 °C for 30 min, and (ii) rinsing three times with distilled water, until a neutral pH is reached [33,34,35]. Finally, the membrane was washed with nitric acid (pH = 2) and rinsed again before measuring water permeability to assess membrane regeneration, with a focus on its temporal variation during multiple filtration and cleaning cycles. This evaluation was based on the conventional fouling characterization approach, where a reduction in permeability during a filtration cycle is typically attributed to short-term fouling phenomena. These include particle deposition on the membrane surface or pore blockage, particularly in the context of UF applications. The initial water permeability through 150 and 50 kDa membranes was 180 and 80 L h^−1^ m^−2^ bar^−1^, respectively. After each cleaning step, this initial membrane permeability was recovered.

#### 2.2.4. Analytical Methods

Pretreated and treated OMW were analyzed for physicochemical characteristics to monitor changes in pollutant concentration throughout the various treatment processes. pH and turbidity were measured using a pH meter type HACH sensION + pH3 (Loveland, CO, USA) and a turbidimeter type WTW Turb 55Q IR (Loveland, CO, USA). Conductivity was assessed with a WTW conductivity meter. COD was determined using a photolab 7100-Vis spectrophotometer (San José, Canada). TOC was measured using a TOC-L type total organic carbon analyzer (SHIMADZU, Kyoto, Japan).

#### 2.2.5. Phytotoxicity Test

Since there is a direct correlation between COD and the toxicity of wastewater, it is essential to use both bioassay toxicity tests and physicochemical analyses to evaluate the risks represented by pretreated and treated wastewater discharges towards the environment, or their reuse in irrigation. Kasmi et al. [36] noted that the organic content of OMW is characterized by high concentrations of BOD_5_ (exceeding 100 g O_2_ L^−1^) and COD (greater than 220 g O_2_ L^−1^) and a strong unpleasant odor. This effluent contains phenolic compounds, which are the most recalcitrant fraction and contribute significantly to the phytotoxic effects of OMW. These effluents have been shown to negatively impact plant growth, causing delays in germination and hindering plant development, primarily due to elevated concentrations of phenolic compounds and heavy metals, particularly when the solutions are not diluted. Additionally, salinity, caused by excess sodium (Na^+^) and chloride (Cl^−^) ions, represents a significant constraint on plant production, adversely affecting plant growth and development. Bioassays are widely regarded as a standard method for assessing the toxicity of substances or complex mixtures. These tests are valuable tools for evaluating the toxic effects of the diverse mixtures often present in industrial effluents. In fact, physicochemical parameters alone are insufficient to capture the harmful impacts of industrial wastewater on aquatic or terrestrial organisms. This underscores the importance of bioassay tests as reliable procedures for assessing the toxicity of the complex mixtures in industrial wastewater. Furthermore, toxicity tests are effective tools for demonstrating the efficacy of wastewater treatment processes in removing toxic substances. Many plant species have been proposed as biomarkers for assessing terrestrial toxicity, with root elongation of germinated seeds being a common method. Among these, lettuce seeds (*Lactuca sativa*) have been specifically recommended by the U.S. Environmental Protection Agency (1982) as an effective indicator. Lettuce is an ideal choice for demonstrating the potential adverse effects of using wastewater for irrigation. In our study, the phytotoxicity test using *Lactuca sativa* seeds is the most suitable bioassay. To evaluate the phytotoxicity of various raw and pretreated and treated wastewater samples, the germination index (GI) of *Lactuca sativa* was calculated following the methodology outlined by Tiquia (2000) [17]. Ten seeds were evenly placed in Petri dishes containing Whatman filter paper, and 10 mL of wastewater samples were carefully added to each dish. The dishes were then covered and placed in a dark incubator at a temperature of 20 ± 2°C for a period of 7 days. Distilled water and drinking water were used as the control. Phytotoxicity tests were conducted in duplicate for each sample, and the average values were recorded. After the incubation period, the number of germinated seeds was counted, and the results were expressed as relative seed germination (RSG), relative root growth (RRG), and the germination index (GI) of lettuce seeds, which are calculated according to [17]:

RSG (%) = (number of germinated seeds/number of seeds) × 100 (1)

RRG (%) = (relative root length of the germinated seeds in the sample/relative root length of the germinated seeds in distilled water). (2)

GI = (RSG × RRG)(3)

#### 2.2.6. Adsorption on Activated Carbon (AC)

Adsorption experiments were carried out for evaluating the suitable mass of AC for optimal OMW treatment, based on turbidity, COD, and TOC removal efficiencies. The experiments were carried out on raw, pretreated, 0.45 µm DEF permeate OMWs, which were mixed with different masses of AC (C1220G95 in grains mesh, Carbio12, Carnoux en Provence, France) at a speed of 400 rpm for 72 h. 100 mL of raw and permeate samples were mixed separately with 2, 4, 10, 20, 50, and 100 g AC L^−1^.

## 3. Results

### 3.1. Treatment of OMW Using DEF Membrane

#### Influence of MWCO

**Figure 3:**
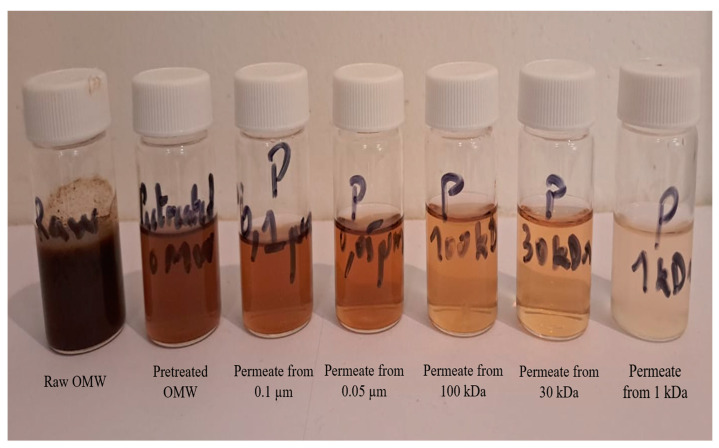
Raw, pretreated OMWs and permeates from the different MWCOs.

The impact of membranes’ MWCO on permeate quality was investigated to assess the performance and contaminant removal efficiency of various membranes. Specifically, the removal of COD, TOC, and turbidity was evaluated, as these parameters primarily reflect the removal of organic matter [17]. The experiments utilized pretreated OMW, which was characterized by a high organic load, with an initial COD of 10,960 mg O_2_ L^−1^, a TOC of 971 mg L^−1^, and a turbidity of approximately 371 NTU. For microfiltration (MF) membranes with pore sizes of 100 to 0.1 µm, the removal efficiencies varied as follows: COD removal was around 80%, TOC removal was around 10%, and turbidity removal ranged from 36.9% to 99%. Despite these removal rates, the residual concentrations of COD, TOC, and turbidity in the permeate remained high, ranging from 2610 to 1950 mg O_2_ L^−1^ for COD, 924 to 858 mg L^−1^ for TOC, and 234 to 1 NTU for turbidity. These COD values remain above the discharge limit of 0.5 g O_2_ L^−1^ set by the Moroccan standard (Official Bulletin No. 6199). It is important to note that the variation in COD and TOC is constant whatever the cut-off, and that turbidity of the order of 4.17 and 1 NTU is obtained for a cut-off of 0.45 and 0.1 µm. This improvement is attributed to the smaller pore sizes, which enhances their retention and selectivity [17]. UF membranes demonstrated a similar performance, even though the MWCO was decreased to 1 kDa. Indeed, turbidity dropped to less than 1 NTU and the obtained COD and TOC values showed removal efficiencies of around 85% and 42%, respectively. Table 2 and Figure 3 show the main results. It is important to note that the percentage of elimination is calculated relative to the effluent obtained after pre-treatment. Compared to the initial OMW, these percentages are higher.

For low MWCO, a small volume (50 mL) of the effluent was filtered and for larger MWCO, the DEF filtrations were conducted in semi-continuous mode using 0.45 µm and 0.22 µm membranes, with the permeate flux being monitored as a function of the volumic concentration Factor (FCV) in the cell filtration (50 mL). The permeate flux through the 0.45 (0.22) µm membrane strongly decreased from 69.2 (61) to 27 (21.5) kg h^−1^ m^−2^, then it stabilized around this value until the end of filtration, corresponding to an FCV value of 5.2. TOC drops very slowly, while COD drops immediately and then remains almost constant. High MWCOs retain several organic compounds, but they are not sufficiently selective for other compounds such as polyphenols that pass into the permeate. As a result, the TOC of the permeate remains close to that of the feed, especially if the concentration of these polyphenols is high (i.e., high TOC), which is the case for the pretreated OMW. As the MWCO decreases sharply, polyphenols are increasingly retained, leading to a subsequent decrease in TOC.

### 3.2. Evaluation of the Phytotoxicity of Treated OMW

**Figure 4:**
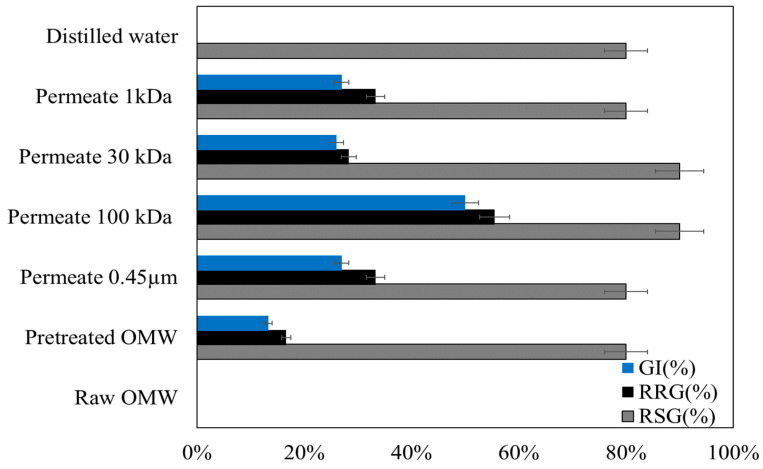
Germination indexes of *Lactuca sativa* seeds under raw and treated OMWs (permeates) using DEF.

Despite integrated treatment, OMW can still present environmental toxicity. In addition to evaluating physical and chemical indicators, toxicity tests are commonly used to assess the degree of wastewater toxicity [36,37]. In this study, the seed germination of lettuce (*Lactuca sativa*) was employed to assess the feasibility of reusing treated wastewater for irrigation. The seed germination index (GI) serves as an effective indicator of phytotoxicity [38,39]. The phytotoxicity of raw and treated OMW using the DEF was evaluated by measuring and calculating the relative seed germination (RSG), relative root growth (RRG), and GI of lettuce seeds over a 7-day period (Figure 4).

The results revealed that the GI for raw OMW was 0%. A GI greater than 50% is considered non-phytotoxic, while a GI below 50% is considered phytotoxic [40,41]. These results indicate high phytotoxicity in raw OMW [42,43]. However, seeds irrigated with treated OMWs using DEF exhibited higher germination rates compared to those irrigated with raw wastewater. Despite this finding, no significant beneficial effect of membrane filtration on seed germination in *Lactuca sativa* was observed (GI under 50%), except for the 100 kDa filtration. Based on the GI, the 100 kDa DEF was the most effective in removing the toxicity of the OMW, yielding results comparable to those of distilled water. Additionally, in conjunction with the GI, the RSG and RRG were analyzed to assess the effects of raw and treated OMWs on seed germination and root growth in lettuce. RSG and RRG improved from 80% to 90% and from 16% to 55%, respectively, in contrast to the values observed in raw OMW (0% for both RSG and RRG). The RSG for OMW treated with 30 kDa and 100 kDa membranes was similar, as was the RSG for all MWCOs permeates and distilled water/control. Based on these findings, the phytotoxicity assessment of OMW indicates that raw OMW inhibits seed germination and growth in *L. sativa*. Therefore, the direct application of raw OMW in agricultural fields is not recommended, but in contrast, treatments using ultrafiltration processes are suitable for agricultural applications without requiring additional precautions. Based on the results, a cut-off value of 150 kDa was selected due to a removal rate higher than 99.8% 80%, and 30%, respectively, for turbidity, COD, TOC, and phytotoxicity. Utilizing a lower MWCO is considered unnecessary, as it would result in a reduced permeate flux without significantly enhancing the retention of pollutants. Although a UF membrane is employed, it is chosen specifically for its ability to provide higher retention rates compared to MF membranes, particularly during the effluent concentration phase.

### 3.3. Treatment by Adsorption on Activated Carbon

**Figure 5:**
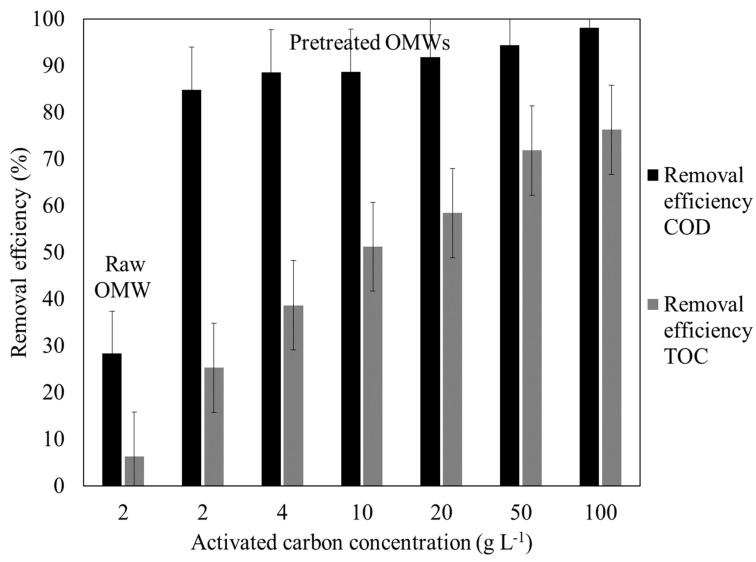
Removal efficiencies for COD and TOC of different OMW samples treated by adsorption on AC.

Adsorption on AC was also carried out on jar testing to compare its removal efficiencies with the DEF filtrations. Figure 5 shows the evolution of these parameters as a function of the AC concentration added to each OMW sample.

Raw OMW treatment resulted in a reduction of 28.31% and 6.31% for COD and TOC, respectively, as the sample was highly loaded with organic matter and the utilized AC mass was low. As depicted in Figure 5, COD and TOC removal efficiencies reached approximately the same values (85 and 35%) for pretreated OMW samples (which were mixed with large AC concentrations of 2 g L^−1^) as those for the 100 kDa DEF. The parameters for COD and TOC increased once the AC concentrations increased to very high concentrations ranging from 2 g L^−1^ to 100 g L^−1^, reaching maximum values of 98% and 76%, respectively (even though this carbon dose is not feasible in practical industrial applications) [44].

### 3.4. Cross-Flow Ultrafiltration

**Figure 6:**
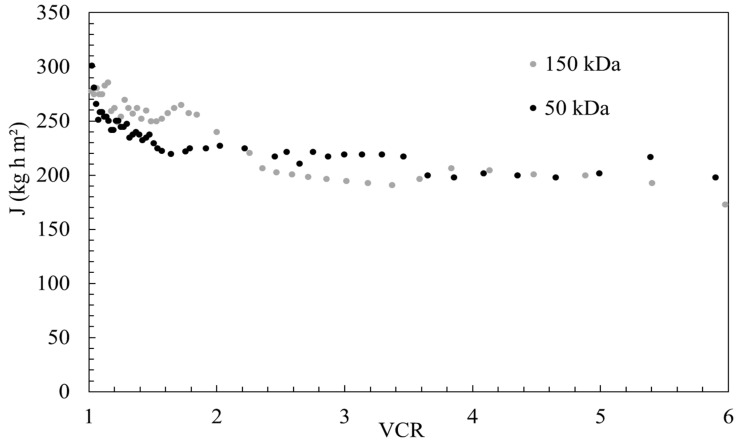
Variation in permeate flux as the function of volume concentration ratio (VCR) for 150 kDa and 50 kDa cross-flow UF.

The pilot-scale unit was operated in batch mode, wherein the retentate was completely returned to the feed tank for recycling through the module to treat the pretreated OMW. As the operation progressed, the COD and TOC concentrations in the feed tank gradually increased. Due to the operational constraints of the pilot plant, a specific volume concentration ratio (VCR) was established as the endpoint of the batch experimental runs, in alignment with the industrial objectives. The effect of membrane filtration as a post-treatment process during the reclamation of treated OMW using a cross-flow UF process was evaluated. Specifically, the permeate from the OMW treatment, using a 150 kDa membrane cut-off, was subjected to a subsequent CFF process using a 50 kDa membrane. The 150 kDa membrane was employed, as the permeate from the 100 kDa DEF delivered the highest GI while the 50 kDa membrane was also used for the post-treatment, based on the 30 kDa DEF performance in turbidity, COD, and TOC removal rates (99.8%, 85%, and 42%, respectively). The use of a 1 kDa membrane could probably have reduced the permeate flux and extended the filtration time. In addition, the increase of the permeate flux through a 1 kDa membrane requires a larger membrane surface, which leads to a higher operational cost in the semi-industrial (this study) or in the industrial scale. Partial (COD and TOC) and overall mass balances were close at less than 10–1% and 16–2% for 150 and 50 kDa, respectively. The permeate flux through the 150 (50) kDa membrane decreased from 278 (301) to 215 (91) kg h^−1^ m^−2^, at a VCR value of 2, corresponding to half the OMW feeding mass. (Figure 6).

**Figure 7:**
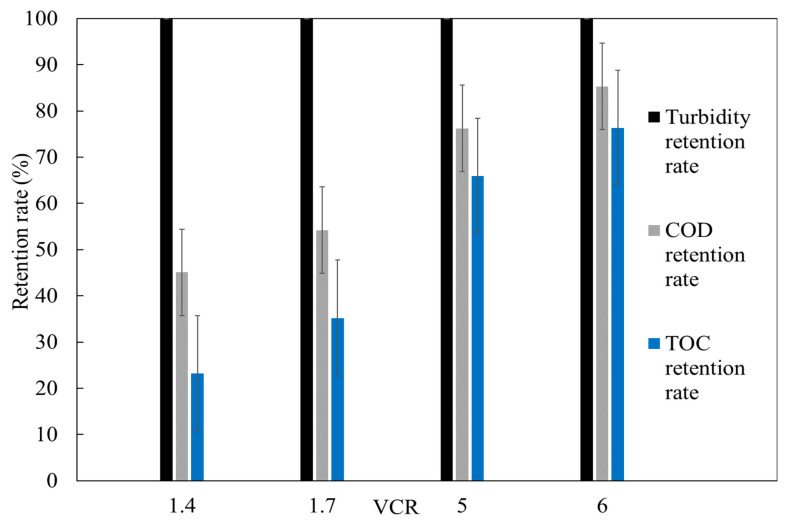
Variation in turbidity, COD, and TOC retention rates for 150 kDa UF.

Regarding turbidity removal efficiency, no significant differences were observed throughout the entire treatment process, with removal values consistently exceeding 99%. However, as the feed suspensions were concentrated, there was a slight increase in the permeate’s COD and TOC concentrations, with retention rates ranging from 45% to 86% for COD and from 23% to 78% for TOC (Figure 7).

**Figure 8:**
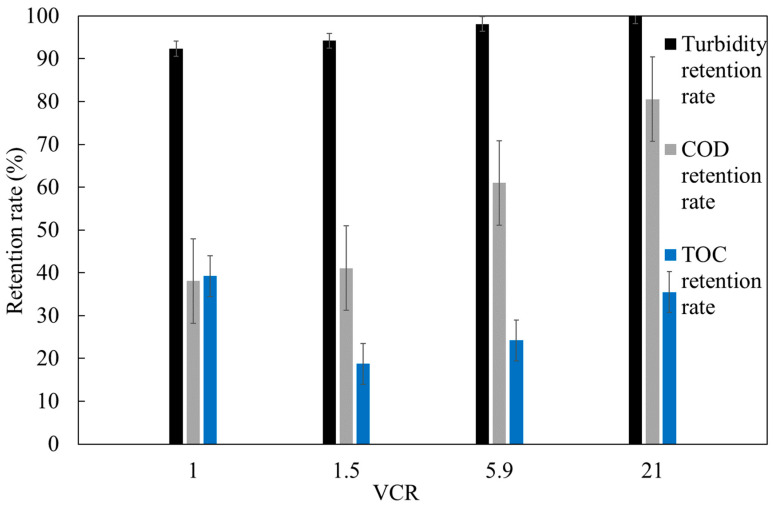
Variation in turbidity, COD, and TOC retention rates for 50 kDa UF.

The cross-flow UF treatment of OMW demonstrated a recovery rate of 84% for treated water. Additionally, high pollutant retention was achieved, with average residual turbidity around 1 NTU (corresponding to a 99% turbidity removal), residual COD at approximately 2655 mg O_2_ L^−1^ (86% average COD retention rate), and residual TOC at about 1151 mg L^−1^ (78% average TOC retention rate). Nevertheless, the COD levels in the treated OMW remained significantly above the discharge limit of 0.5 gO_2_L^−1^, as specified by the Moroccan standard (Official Bulletin No. 6199). To enhance permeate quality or membrane permeability, it is recommended to implement (a) a pretreatment step prior to the UF process, such as CWs, and/or (b) a post-treatment filtration process for the permeate. An additional ultrafiltration with a MWCO of 50 kDa was carried out in order to estimate whether it was possible to reduce the TOC and COD of the final permeate. The post-treatment with the membrane had a significant impact on COD removal efficiency from pretreated OMW (78.5%). As anticipated, pollutant rejection increased as the MWCO decreased. COD retention rates increased from 38% to 84%, with the initial COD concentration around 2655 mgO_2_ L^−1^. Similarly, TOC retention rates increased from 39% to 46%, for an initial TOC concentration of approximately 1151 mgL^−1^. Furthermore, the tested membrane demonstrated excellent turbidity retention, with rates ranging from 92% to 99.9%, producing permeates with residual turbidity levels below 1 NTU (Figure 8).

## 4. Conclusions

Cross-flow ultrafiltration (UF) of pretreated olive mill wastewater (OMW) using ceramic tubular membranes has emerged as a promising treatment method. In this work, it was possible to comprehend the impact of this separation technique on the depollution of this wastewater. Different molecular weight cut-offs (MWCO) in dead-end filtration (DEF) of pretreated OMW were tested, and a cut-off value of 150 kDa was selected due to a removal higher than 99.8%, 80%, and 30%, respectively, for turbidity, COD, and TOC, and a low phytotoxicity. Adsorption of raw and pretreated OMW on granular activated carbon (AC) was also carried out for a comparative study against DEF and pretreatment. COD and TOC removal efficiencies reached maximum values of 98.1% and 76.2%, respectively, but for a large concentration of 10 g L^−1^. The UF of pretreated OMW through the 150 kDa membrane achieved the highest permeate flux of 277 L h^−1^ m^−2^ at a constant TMP of 3.3 bars. Regarding turbidity retention rates, no significant differences were observed throughout the entire treatment process, with values exceeding 99%. COD and TOC retention rates reached maximum values of 86% and 78%, respectively. In order to enhance the permeate quality and the global COD and TOC removal efficiency from the pretreated OMW, the permeates obtained from the 150 kDa membrane were subjected to a post-CF UF through a smaller MWCO (50 kDa) membrane. The permeate flux reached a value of 301 kg h^−1^ m^−2^ at a TMP of 3.8 bars and a permeate flow rate of 46 L h^−1^. This post-treatment had a significant impact on COD removal efficiency from pretreated OMW, which dropped to 78.5%. COD and TOC retention rates reached maximum values of 83.7% and 46.1%, respectively, while turbidity retention reached 99.9%. Finally, the coupled process proposed in this study removes 97%, 97%, and 99.9% of COD, TOC, and turbidity, respectively. The results underscore that membrane performance is heavily influenced by the organic load of the wastewater, with the interaction between the feed components and the membrane material playing a key role. These findings also highlight the importance of selecting the appropriate membrane and operating conditions based on the characteristics of the feed solution. Furthermore, the CW pre-treatment was effective in reducing membrane fouling and delivering wastewater that did not affect the water permeability through the membrane after each ultrafiltration. This coupled CW/UF process is a promising solution for water resource recovery and pollution removal, although it does not produce a treated liquid with a COD concentration under the discharge limit. Overall, these findings can be valuable for the development and optimization of industrial systems designed to treat and reuse the olive mill wastewater.

## Figures and Tables

**Table 1:** Physico-chemical characteristics of the settling tank effluent and VFCW units’ efficiencies in the reduction of organic matters as well as nutrients.

Parameters	Influent	Settling Tank Effluent	RR (%)	Unplanted Unit						Planted Unit					
				Min	RR (%)	Max	RR (%)	V	SD	Min	RR (%)	Max	RR (%)	V	SD
pH	5.02	4.9	------	4.71	---	4.86	----	0.002	0.04	4.74	-----	5.01	----	0.001	0.11
Temperature (°C)	20.65	20	------	20	---	20.23	----	0.009	0.09	20	---	21	---	0.354	0.59
Conductivity (µS cm^−1^)	1530	1500	------	1423	----	1433	----	40.46	6.36	1462	----	1475	----	87.7	9.36
COD (mg O_2_ L^−1^)	101,700	50,850	50	1433	97	15,342	69	1876	4333	10,960	78	12,013	76	2554	1598
TSS (mgL^−1^)	54,605	25,233	53	18,342	27	20,564	18	466	682	12,616	50	13,343	47	59	243
BOD_5_ (mg O_2_ L^−1^)	41,697	22,099	47	6637	70	7653	65	124	353	4055	81	6487	70	5297	2302
TOC (mg L^−1^)	33,498	17,118	48	1323	92	1400	91	578.3	24	971	94	1100	93	2762	52.5
Turbidity (NTU)	1700	1020	40	493	51	522	48	134.2	11.6	371	63	480	52	2365	48.6
NH_4_^+^ (mg L^−1^)	341	341	----	46	86	58	83	72.15	8.49	36	89	42	87	3.73	1.93
PO_4_^3−^ (mg L^−1^)	919	919	-----	156	83	170	81	20.23	4.5	99	89	124	86	63.97	8

V: variance, SD: standard deviation.

**Table 2:** DEF filtrations performances.

Sample	pH	MAE	Conductivity (µS cm^−1^)	MAE	Turbidity (NTU)	MAE	Turbidity Removal Efficiency (%)	COD (mgO_2_ L^−1^)	MAE	COD Removal Efficiency (%)	TOC (mg L^−1^)	MAE	TOC Removal Efficiency (%)
Permeate 100 µm	4.92	0.09	1494	4	234	0.7	36	2610	6.67	76	924	6.67	4
Permeate 8 µm	4.95	0.07	1496	4	79.7	0.2	78	2050	1.33	81	863	2	11
Permeate 5 µm	4.83	0.11	1495	3.33	76.1	0.07	79	2030	5	81	858	1.33	11
Permeate 0.8 µm	4.91	0.06	1495	3.33	74.5	0.33	79	1950	3.33	82	862	1.33	11
Permeate 0.45 µm	5.02	0.04	1488	5.33	4.17	0.05	98	2450	3.33	77	932	6.67	
Permeate 0.22 µm	4.85	0.03	1503	4.67	1.18	0.05	99	2230	1.33	79	915	6.67	
Permeate 0.1 µm	4.85	0.03	1501	2.67	1	0	99	2220	13.33	79	861	3.33	11
Permeate 0.05 µm	4.87	0.02	1508	2	0.78	0.02	99	2085	6.67	81	859	0.67	11
Permeate 100 kDa	4.89	0.03	1493	2	0.67	0.02	99	1823	0.67	81	645	1.33	33
Permeate 30 kDa	4.89	0.03	1483	2	0.5	0.07	99	1600	0.67	85	566	0.67	41
Permeate 1 kDa	4.87	0.02	1465	3.33	0.34	0.03	99	1300	1.33	88	460	2	52

## Data Availability

The original contributions presented in this study are included in the article. Further inquiries can be directed to the corresponding author.

## Abbreviations

AC	Activated carbon
BOD_5_	Biochemical oxygen demand (mg O_2_ L^−1^)
CFF	Cross-flow filtration
COD	Chemical oxygen demand (mg O_2_ L^−1^)
CWs	Constructed wetlands
DEF	Dead-end filtration
VCF	Volumic concentration factor
FO	Forward osmosis
FWSF	Free water-surface flow
GI	Germination index (%)
MF	Microfiltration
MWCO	Molecular weight cut-off (Da)
OMW	Olive mill wastewater
RR	Removal rate (%)
RRG	Relative root growth (%)
RSG	Relative seed germination (%)
SS	Suspended solids (mg L^−1^)
SSF	Subsurface flow
TMP	Trans-membrane pressure (bar)
TN	Total nitrogen (mg L^−1^)
TOC	Total organic carbon (mg L^−1^)
TP	Total phosphorus (mg L^−1^)
TS	Total solids (mg L^−1^)
TSS	Total suspended solids (mg L^−1^)
UF	Ultrafiltration
VCR	Volume concentration ratio
